# You are what you eat: How to best fuel your immune system

**DOI:** 10.3389/fimmu.2022.1003006

**Published:** 2022-09-20

**Authors:** Charlotte Hellmich, Edyta E. Wojtowicz

**Affiliations:** ^1^ Norwich Medical School, University of East Anglia, Norwich Research Park, Norwich, United Kingdom; ^2^ Department of Haematology, Norfolk and Norwich University Hospitals NHS Trust, Norwich, United Kingdom; ^3^ Earlham Institute, Norwich Research Park, Norwich, United Kingdom

**Keywords:** bone marrow, hematopoiesis, diet, metabolism, infection

## Abstract

Normal bone marrow (BM) homeostasis ensures consistent production of progenitor cells and mature blood cells. This requires a reliable supply of nutrients in particular free fatty acids, carbohydrates and protein. Furthermore, rapid changes can occur in response to stress such as infection which can alter the demand for each of these metabolites. In response to infection the haematopoietic stem cells (HSCs) must respond and expand rapidly to facilitate the process of emergency granulopoiesis required for the immediate immune response. This involves a shift from the use of glycolysis to oxidative phosphorylation for energy production and therefore an increased demand for metabolites. Thus, the right balance of each dietary component helps to maintain not only normal homeostasis but also the ability to quickly respond to systemic stress. In addition, some dietary components can drive chronic inflammatory changes in the absence of infection or immune stress, which in turn can impact on overall immune function. The optimal nutrition for the best immunological outcomes would therefore be a diet that supports the functions of immune cells allowing them to initiate effective responses against pathogens but also to resolve the response rapidly when necessary and to avoid any underlying chronic inflammation. In this review we discuss how these key dietary components can alter immune function, what is their impact on bone marrow metabolism and how changes in dietary intake of each of these can improve the outcomes of infections.

## Introduction

Haematopoiesis is a tightly controlled process that occurs in the specialized bone marrow (BM) niche, consisting of both haematopoietic cells and numerous support cells, including BM stromal cells, adipocytes, endothelial cells, osteocytes and osteoblasts (1). Not only does the environment support the maintenance of self-renewing haematopoietic stem cells (HSCs) and the production of mature haematopoietic cells during normal haemostasis, but it is also required to rapidly adapt and respond to local and systemic stressors. This includes the rapid expansion of HSCs and progenitor cells and increased production and mobilization of mature immune cells in response to infection (2, 3). Bacterial infections, for example, trigger the release of granulocyte stimulating factor (G-CSF) from endothelial cells to drive emergency granulopoiesis in order to meet the increased demand for neutrophils (4, 5). In addition some bacterial infections drive production of monocyte chemoattractant protein 1 (MCP-1), a chemokine that activates the CCR2 receptor on monocytes and has been shown to be essential for the mobilization of monocytes out of the BM (6). Other chemokines and cytokines such as Interleukin 6 (IL-6) and interferon gamma (IFN-γ) have also been implicated in driving the myeloid expansion in response to infections (7, 8). Together, emergency myelopoiesis results in a neutrophilia, with release of immature (left shifted) neutrophils and a monocytosis in the peripheral blood (9). This forms part of the immediate immune response to an acute infection and helps to compensate for the increased consumption of myeloid cells at the site of infection. The BM response to viral infections is less well understood and may in some ways be more complex (10). Lymphocytes, particularly T cells and natural killer cells are often key to the clearance of viral infections and for many an adaptive immune response is required for complete clearance of the virus (11). Furthermore, viral infections can have more long-term consequences for BM cell populations and HSC health (12) and have been implicated in causing cytopenias, aplastic anaemia as well as being associated with lymphoid BM malignancies (13–15).

In order to facilitate this rapid and efficient expansion of mature immune cells, HSC and progenitor cells require adequate metabolic supplies and are able to utilise different pathways to maximise their energy production. The HSC niche is a hypoxic environment and in the steady state HSCs primarily rely on glycolysis (16). However, in response to stress they are able to rapidly switch to oxidative phosphorylation (2, 17). This change in energy production is partially driven by their individual requirements but also affected by the energy sources available to the cells. Here we will discuss how alterations in diet influence the metabolic processes involved in the BM response to stress. Here we will focus on the macronutrients including carbohydrates, fatty acids and protein as these have been extensively studied.

## Dietary components and their impact on immune function

### Fatty acids

Fatty acids can be classified by their structure (saturated or unsaturated) or length (short-chain, medium-chain and long-chain). The different groups of fatty acids vary in their roles and functions in different organs and consequently can influence the immune system in different ways. For example imbalance between saturated and unsaturated fatty acids has been shown to contribute to the aetiology of allergic, autoimmune and metabolic diseases (18–20). Western diets are rich in sugar, trans and saturated fats, but low in complex carbohydrates, fiber, micronutrients, polyphenols and omega 3 polyunsaturated fatty acids (PUFA’s). This diet has been linked to an increased uptake of LPS (lipopolysaccharide) from microbes in the gut due to increased gut leakiness (21). LPS stimulates innate immune cells and activates them *via* Toll-like receptor (TLR) 4) contributing to chronic inflammation manifested by elevated serum levels of proinflammatory cytokines (e.g. IL-1, IL-6, IL-8, IL-12, and TNFα), chemokines (e.g. MCP-1, RANTES, and MIP-1) and acute phase reactants (e.g. C-reactive protein, serum amyloid A, and ferritin) (22, 23). On the contrary, omega 3 and PUFAs can interfere with TLR4 activation through inhibition of COX-2 mediated prostaglandin release and thus ameliorate this inflammatory signal (24, 25).

Furthermore, it has been shown that n=3 PUFA’s can increase the phagocytic activity and inhibit apoptosis in alveolar macrophages (26, 27).

**Figure 1:**
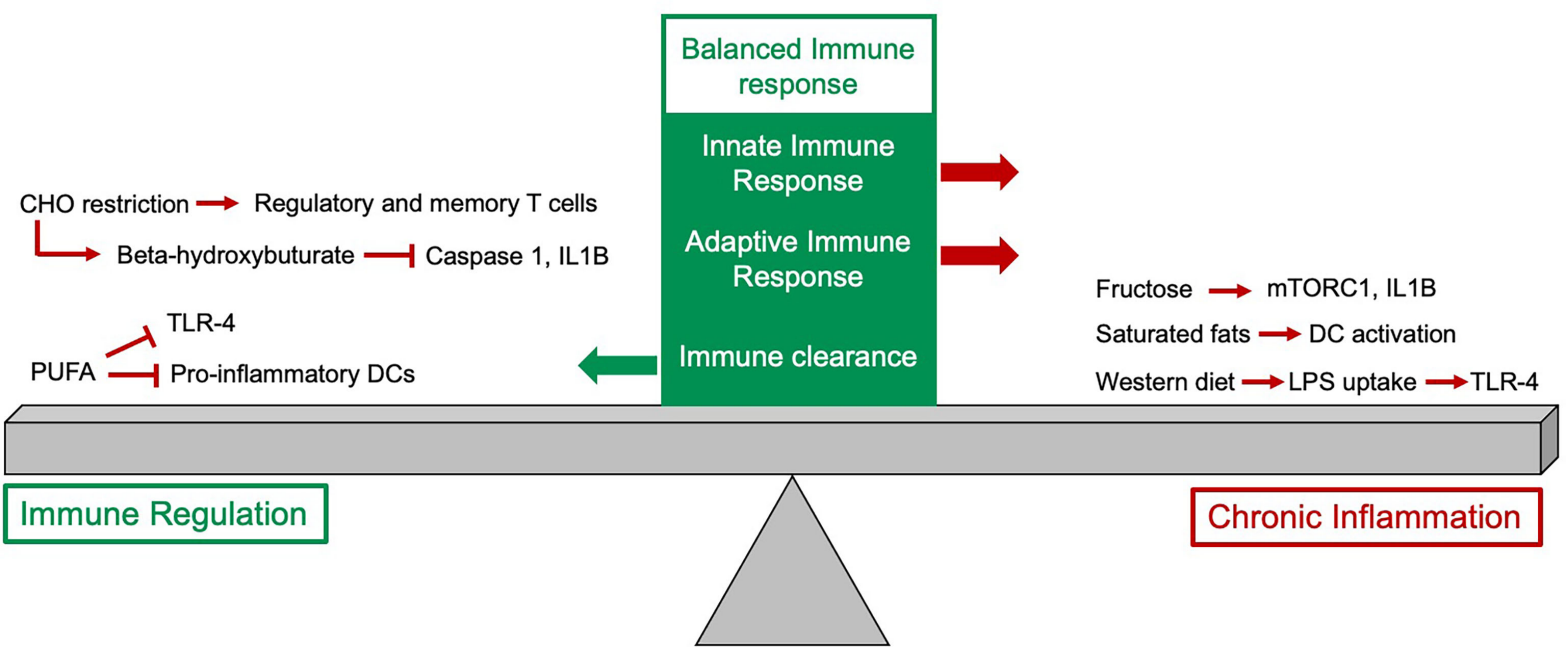
The impact of diet on the immune response. The balanced immune response is dependent on expansion of innate and adaptive immune cells as well as an appropriate downregulation of the response. Some dietary components, particularly fructose, saturated fats and the typical western diet have pro-inflammatory effects and if these persist this can drive chronic inflammation. Other dietary changes, such as reduction in carbohydrate (CHO) intake and increased intake of polyunsaturated fatty acids can help to downregulate the immune response.

PUFA’s also have an inhibitory effect on the pro-inflammatory phenotype of dendritic cells (DC) and DC-mediated T cell responses (28, 29). In contrast, saturated fatty acids increase DC maturation, activation and T-cell stimulation properties (30). Thus, whilst common fat components of the typical western diet can drive an unwanted chronic inflammatory process, other fatty acids play a key role in regulating the immune response and reducing tissue inflammation (**Figure 1**).

### Carbohydrates

Activation of the innate immune system requires metabolic rewiring primarily to favor oxidative glucose metabolism to activate macrophages and propagate their adhesion (31–34). In this scenario glucose is the major source of carbon, however increased blood levels of fructose present in Western diets (35) metabolically rewire innate immunity. In the recent study monocytes and macrophages exposed to fructose (main component of broadly used corn syrup) have shown increased activity of mTORC1 and expression of IL1B in response to LPS. Furthermore, these cells have reduced metabolic flexibility in response to glycolytic and mitochondrial inhibition, underpinning the pro-inflammatory role of dietary fructose (36).

B and T cells constitute major pillars of adaptive immunity. It has been shown that glucose is a major driver of B cell development and function.Increasing glucose levels drive expansion of splenic and mesenteric lymph node and Peyer’s patch B cells, which was also linked with enhanced B-lymphopoiesis in the bone marrow. Importantly, these B cells had increased immunoglobulin-G production after immunization and reduced apoptosis levels in early development *via* mTOR activation (37). Interestingly, human breast milk contains 7% carbohydrates (87% water, 1% protein, 4% lipid), which therefore forms the biggest nutrient resource for breast milk fed infants (38). As high carbohydrate intake has been shown to drive B cell development it is possible that this is the driver for B cell HSC skewing in infants (39). On the other hand, restriction of carbohydrate intake leads to production of ketone bodies such as beta-hydroxybutyrate (BHB), which can be utilized *via* mitochondrial oxidative phosphorylation (40). In animal models, BHB has been shown to attenuate caspase-1 activation and IL1B production by inhibiting NLRP3 inflammasome in phagocytes thus ameliorating low-level inflammation and associated autoinflammatory diseases (41–43). Interestingly, in human volunteers on a very-low-carbohydrate diet CD4+, CD8+ and regulatory T-cell capacity was markedly enhanced and T memory cell formation was increased. Molecular analysis of these cell subsets revealed an immunometabolic reprogramming in response to ketones favoring oxidative metabolism and conferring superior cellular energy supply and reactive oxygen species (ROS) signaling (44). The intake and availability of carbohydrates can therefore not only alter the inflammatory profile associated with both the innate and adaptive immune response but also the balance between different leukocyte populations produced and how they function (**Figure 1**).

### Protein/amino acids

Activation of immune cells relies on specific amino acids, which are signaling molecules or provide metabolites (for TCA) and are not only involved in protein synthesis.Activated T cells upregulate amino acid transporter expression (45–47) required for rapid proliferation, translation of critical cytokines and adhesion molecules. CD4 and CD8+ T cells have similar repertoire of nutrient transporters, however differ in their expression level, where CD4+ have less copies of them which can underlie lower proliferative capacity compared to CD8+ T cells (47, 48). In addition innate immune cells, particularly activated macrophages, heavily depend on arginine uptake *via* CAT2 (49), while resting macrophages likely rely on CAT1 (50) and glutamine (51). LPS increases Slc7a5 transporter expression and leucine uptake to support proinflammatory macrophage cytokine production (52). NK cell activation depends on glutamine acquisition (53), while glutamine metabolism supports antibody production by activated B cells (54).

Glutathione (GSH) is a small molecule composed of glycine, glutamate and cysteine and is detrimental for quenching ROS upon T cell activation (55) or IL1B mRNA synthesis in macrophages stimulated with LPS (56). Branched amino acids (BCAAs)-leucine, isoleucine and valine provide coenzyme A (CoA) derivatives, which support metabolic reprogramming of immune cells (57), activate mTORC1 pathway (58) and epigenetic modification (acetylation) in macrophages or CD8+ T cells (58–60). BCAA by stimulating glucose uptake can promote glycolysis (61, 62).

## Dietary components and their impact on BM metabolism

### Fatty acids

**Figure 2:**
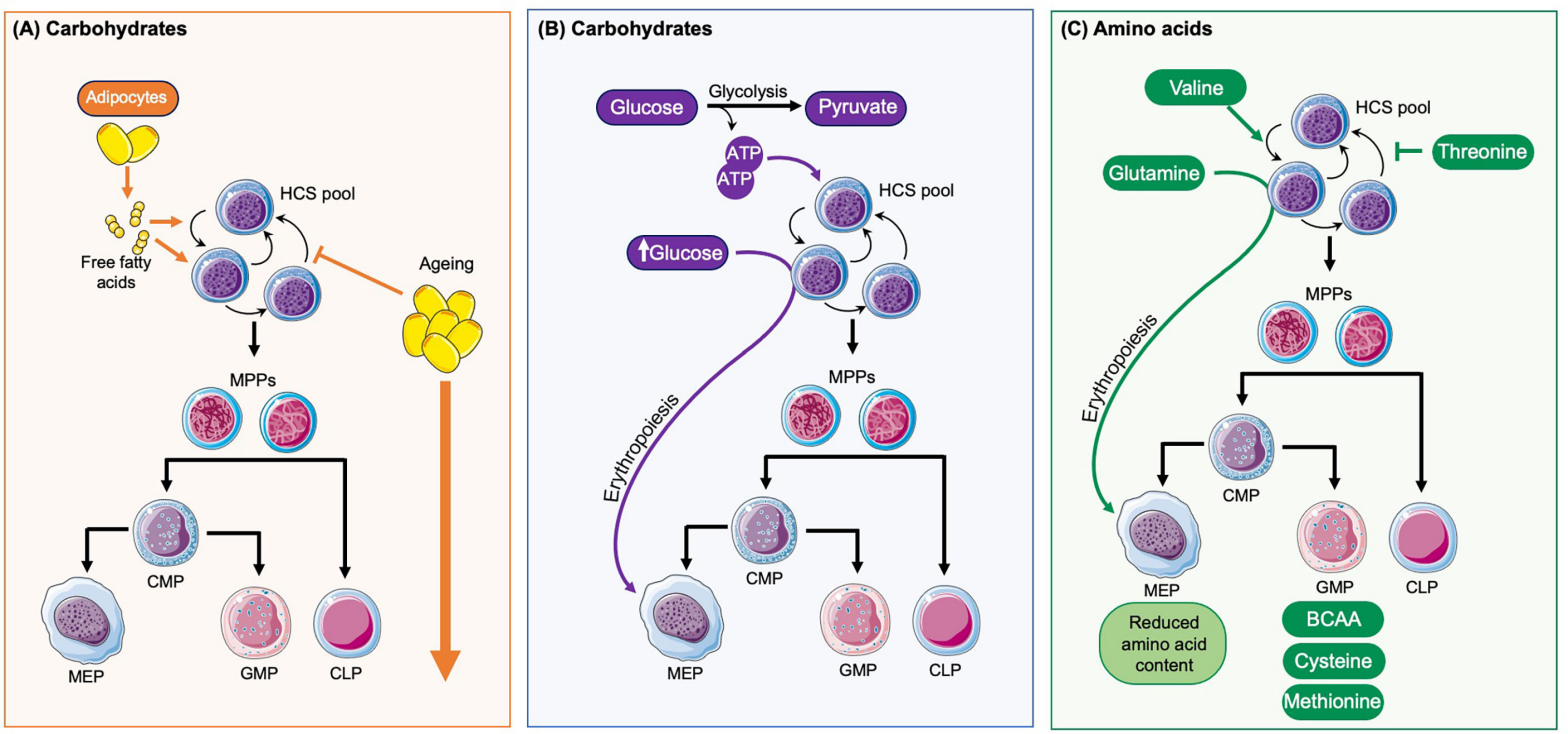
How diet influences haematopoesis. **(A)** Adipocytes are found in close proximity to HSCs and provide free fatty acids, which play a role in HSC maintenance and self-renewal and are an important energy source for HSC expansion. With ageing there is an accumulation of adipocytes which disrupt the normal regulation of haematopoiesis and overall drives the expansion of HSCs and progenitor cells. **(B)** In the steady state HSCs primarily rely on glycolysis for the production of ATP and normal haematopoietic maintenance. High levels of glucose also enhance HSC commitment towards the erythroid lineage. **(C)** The amino acid valine has been shown to be vital for HSC maintenance and self-renewal whilst threonine can disrupt this. Glutamine promotes erythropoiesis. The amino acids cell content varies between different progenitors with MEPs having reduced levels and GMPs the highest levels, particularly of branched amino acids (BRAA), cysteine and methionine.

The BM has vast stores of adipocytes and the proximity of these to the HSC niche is important as adipocytes store and provide free fatty acids (FFA), a crucial metabolite for HSCs and progenitor cells. Fatty acid oxidation has been shown to be essential for healthy HSC maintenance and implicated in self-renewal divisions (63). In response to stress fatty acid oxidation provides further substrates, in the form of acetyl coenzyme A, for the TCA cycle to help facilitate the rapid switch of HSCs from glycolysis to oxidative phosphorylation in response to infection (17). Thus, an adequate and reliable supply of FFA and therefore adipose tissue is required within the BM niche to drive healthy haematopoiesis and support the BM response to infection (**Figure 2A**). However, the adipocyte rich yellow bone marrow expands significantly both in obesity and with increased age (64). This accumulation of adipocytes can disrupt the normal haematopoietic processes and therefore directly impact on the production of mature blood cells in the steady state and the response to infection (65, 66). Increased adipocyte frequency in the BM metabolically reprograms megakaryocytes by active transfer of fatty acids through CD36 and shifts their ploidy towards 32 and 64N (67) and potentially has an impact on platelet activation status (68). Furthermore, obesity does not only drive local changes within the BM microenvironment but the abundance of adipose tissue in other organs have systemic implications, which in turn can alter immune function and BM health. Obesity has been associated with systemic chronic low-level inflammation (69), at least partially mediated by chronic activation of toll TLR 4 (20, 70). This is driven by the secretion of pro-inflammatory cytokines, adipokines and leptins and is known to contribute to many obesity-associated diseases (65, 71). The consequences of this on the BM microenvironment and immune function include increases in haematopoiesis affecting both myeloid and lymphoid lineages (65, 69), and changes in the innate and adaptive immune responses, with disrupted immediate responses observed in the BM (70) as well as impaired memory T-cells responses (72). Together these changes contribute to the increased morbidity and mortality known to be associated with infections in obese people (73, 74). The mechanisms behind these changes are likely multifactorial with both local and systemic factors manipulating the HSC niche, and therefore influencing HSC maintenance, output, and immune overall function. It is possible that the increase in BM cellularity and haematopoiesis reflects an attempt to compensate for the reduced function of immune cells in the obesity-associated environment. A clearer understanding of how the obesity associated pro-inflammatory environment disrupts normal haematopoiesis and the immune response to infection will help inform future treatments of obese patients to promote a better and regulated response to infection.

### Carbohydrates

Dormant HSCs are thought to have low energy consumption when compared to actively dividing cells (75, 76). They reside in the hypoxic BM niche, which is thought to limit oxidative respiration and favor glycolysis (**Figure 2B**) (77). This helps to reduce ROS levels in quiescent HSCs and promotes long-term HSC health. High availability of glucose has been shown to stimulate nucleotide biosynthesis and enhances HSC commitment to erythroid lineage (78). Fasting can exhibit a positive effect on regenerative potential of HSC *via* inhibition of insulin growth factor 1 (IGF1) signaling and decreased their mobilization potential in patients with diabetes type 2 (79).

### Amino acids

Although HSCs have low rates of protein translation suggesting lower amino acid requirement (80), a recent study found that *in vivo* HSC maintenance depends on valine (**Figure 2C**). Dietary depletion of this amino acid in recipient mice for 2 weeks permitted BM transplantation and donor HSC engraftment without irradiation conditioning (81). For *ex vivo* HSC maintenance depletion of threonine (or downstream metabolites) had a positive effect. So far the metabolic function of valine and threonine for HSC maintenance are yet to be determined. Furthermore, HSC commitment to erythroid lineage has been attributed to increased glutamine levels (78). mTor complex 1 (mTORC1) is a central node in metabolic sensing and signaling (82) including essential amino acid leucine, which in turn regulates its activity through GTPase RagA (83). In a recent study RagA was found to be dispensable for HSC function (84), suggesting HSC are resilient to deprivation of certain nutrients.

A comprehensive proteomics study of BM progenitor - lineage- cKit+Sca1+ stem and progenitor cells (LSK), common myeloid progenitors (CMP), granulocytic and monocytic progenitors (GMP) and myeloerythroid progenitors (MEP) - showed differential content of amino acids, with lowest levels detected in MEPs and highest in GMP (in particular BRAA, cysteine, methionine), suggesting their highly proliferative nature compared to other analyzed cell types. On the contrary the global arginine bioavailability ratio (GABR), defined as the ratio between arginine and its major metabolites (ornithine and citrulline), was higher in the LSK and GMP populations. Ex vivo cultures of LKS supplemented with arginine and citrulline were able to expand the proportion of TMRM low cells (85). These metabolites may have a potential to improve *in vivo* HSC function (86, 87). Thus, different amino acids are key to the normal function, maintenance and homeostasis of HSCs and all progenitor cell populations.

## Modulating diet to improve outcome of infection

Both steady state and stress hematopoiesis are dependent on a reliable and easily scalable energy supply. In order to support their varying energy demands, HSCs and progenitor cells are able to adapt and draw upon multiple different metabolites to generate ATP, thus not relying on one sole source of energy within the BM niche.This also means that the source of energy and therefore individual macronutrient dietary components may not necessarily have a direct impact on immune function. For example, it has long been believed that high fat diet content drives not only obesity but also all its associated diseases such as diabetes and heart disease, however more recently this concept has been contested. Studies have demonstrated that high fat diets can be associated with health benefits, these include anti-inflammatory and immune-supportive changes (88, 89). Debates may continue over which macronutrient is superior and drives better health outcomes, however, it is becoming increasingly apparent that a balance of each dietary component is required and that it is not the relative proportions of fat and carbohydrates that drive obesity, disease and immune dysregulation but any tendency to over-eating and excessive calorie intake (90).

In addition to carbohydrates and fat content, there are of course other essential dietary components required for a healthy immune response. These include amino acids, which play a vital role in HSC maintenance and differentiation (78, 85) as well as presenting a further metabolite for oxidative phosphorylation both during emergency haematopoiesis (91) and normal immune cell maintenance (92). For example, glutamine, the most abundant and versatile amino acid, can be used as a substrate for numerous pathways including nucleotide and NADPH synthesis (93) and has also been shown to regulate HSC differentiation (32). During infection or times of stress demand for amino acids increases and inadequate amino acid stores or complete depletion of existing stores, due to longer periods of increased consumption, have been associated with adverse outcomes (36). In contrast to this however, it is becoming increasingly apparent that a low protein diet can have a beneficial impact on the immune response (94) and it is therefore clear that a fine balance is needed to ensure sufficient stores are present to be used when required but any excess is limited to promote long term BM health.

Another factor to consider in the context of diet and the immune response is the effect of the microbiome on haematopoeisis and immune cell production, Previous work has suggested that the absence of commensal microbes can result in defective immune function. This can drive both failure of appropriate immune responses and immune suppression as well as autoimmune conditions (95, 96). Furthermore, there are numerous micronutrients which are key to a healthy immune response and appropriate immune regulation by providing building blocks, modulating cytokine expression and regulating immune cell activity. These include vitamins, some key micronutrients such as vitamins (97, 98), polyphenols, which have been shown to impact on immune cells function and alter expression of pro-inflammatory cytokines (99), and minerals, in particular zinc, where even minor deficiencies result in impaired immune responses which has been shown to be vital for both innate and adaptive immune cell function (100, 101).

An adequate supply of each of the major dietary components, including fat, carbohydrate, and protein, as well as the numerous micronutrients, is required to achieve a sufficient immediate immune response, mount an adaptive immune response with memory components and downregulate or resolve the immune response at the appropriate time to prevent chronic inflammation. In the absence of this the finely regulated BM microenvironment and its ability to mount an effective immune response is disrupted and this will only impact on how the body can respond to an acute infection but will also have long term consequences, with impaired long-term adaptive immunity as well as potentially prolonged periods of inflammation. Deficiencies in the required nutrients can result from any cause of malnutrition, however, when considering the effect of diet on the immune system it is particularly important to consider the aging population. Not only do dietary deficiencies increase with advanced age (102) but it is also known that immune function declines with age (103). It is therefore important to ensure adequate intake of all dietary components for older patients and to consider supplementation of some vital vitamins or minerals, as these have been shown to reduce infections, support the immune response and improve outcomes (104).

## Conclusion

Any immune response is delicately orchestrated to drive the immediate innate reaction, the long-term adaptive response but eventually also to downregulate and control the inflammatory process. This fine balance is controlled by the BM, the BM niche and the circulating mature immune cells. Any disruption of this process will either result in an ineffective immune response, impaired future immune surveillance or state of chronic inflammation which in turn can lead to a number of pathologies. In this review we have demonstrated how different dietary components can impact on each step involved in the immune process. Dietary components are not only important metabolites required for an effective immune response but they also have a number of regulatory functions and therefore can have a much broader systemic impact, which in turn can affect immune function. Nutrition has the potential to effectively treat immune deficiencies related to poor intake, there is a great interest in whether specific dietary interventions can further enhance immune function in sub-clinical situations, and thus prevent the onset of infections or chronic inflammatory diseases.

## Authors contributions

CH and EW conceptualized and wrote the paper. All authors contributed to the article and approved the submitted version.

## Funding

CH is funded by Wellcome Trust Clinical Research Fellowship (220534/Z/20/Z) and was supported by the NNUH charitable fund. EW is funded by a Sir Henry Welcome Postdoctoral Fellowship (213731/Z/18/Z).

## Acknowledgments

The Figures were partly generated using Servier Medical Art, provided by Servier, licensed under a Creative Commons Attribution 3.0 unported license.

## Conflict of interest

The authors declare that the research was conducted in the absence of any commercial or financial relationships that could be construed as a potential conflict of interest.

## Publisher’s note

All claims expressed in this article are solely those of the authors and do not necessarily represent those of their affiliated organizations, or those of the publisher, the editors and the reviewers. Any product that may be evaluated in this article, or claim that may be made by its manufacturer, is not guaranteed or endorsed by the publisher.
